# Public health surveillance of multidrug-resistant clones of *Neisseria gonorrhoeae* in Europe: a genomic survey

**DOI:** 10.1016/S1473-3099(18)30225-1

**Published:** 2018-07

**Authors:** Simon R Harris, Michelle J Cole, Gianfranco Spiteri, Leonor Sánchez-Busó, Daniel Golparian, Susanne Jacobsson, Richard Goater, Khalil Abudahab, Corin A Yeats, Beatrice Bercot, Maria José Borrego, Brendan Crowley, Paola Stefanelli, Francesco Tripodo, Raquel Abad, David M Aanensen, Magnus Unemo, Euro-GASP study group, Jacinta Azevedo, Eszter Balla, Christopher Barbara, Thea Bergheim, Maria José Borrego, Viviane Bremer, Susanne Buder, Panayiota Maikanti-Charalambous, Stephanie Chisholm, Susan Cowan, Brendan Crowley, Tania Crucitti, Mercedes Diez, Mária Dudás, Kirstine Eastick, Agathe Goubard, Maria Haller, Guôrún Svanborg Hauksdóttir, Steen Hoffmann, Gwenda Hughes, Derval Igoe, Samo Jeverica, Irena Klavs, Hilde Kløvstad, Peter Kohl, Vasileia Konte, Ineke Linde, Violeta Mavcutko, Jackie Maistre Melillo, Gatis Pakarna, Peter Pavlik, Despo Pieridou, Guy La Ruche, Guôrún Sigmundsdóttir, Soteroulla Soteriou, Angelika Stary, Paola Stefanelli, Barbara Suligoi, Peter Truska, Eva Tzelepi, Magnus Unemo, Birgit Van Benthem, Alje Van Dam, Julio Vazquez, Inga Velicko, Ruth Verbrugge

**Affiliations:** a Infection Genomics, Wellcome Sanger Institute, Hinxton, UK; b Centre for Genomic Pathogen Surveillance, Wellcome Sanger Institute, Hinxton, UK; c Antimicrobial Resistance and Healthcare Associated Infections Reference Unit, National Infection Service, Public Health England, London, UK; d European Centre for Disease Prevention and Control, Stockholm, Sweden; e WHO Collaborating Centre for Gonorrhoea and Other Sexually Transmitted Infections, Department of Laboratory Medicine, Clinical Microbiology, Faculty of Medicine and Health, Örebro University Hospital, Örebro, Sweden; f Assistance Publique Hôpitaux de Paris, St Louis Hospital, Paris, France; g National Institute of Health, Lisbon, Portugal; h St James's Hospital, Dublin, Ireland; i Istituto Superiore di Sanitá, Rome, Italy; j Carlos III Health Institute, Madrid, Spain; k Big Data Institute, Li Ka Shing Centre for Health Information and Discovery, Nuffield Department of Medicine, University of Oxford, Oxford, UK

## Abstract

**Background:**

Traditional methods for molecular epidemiology of *Neisseria gonorrhoeae* are suboptimal. Whole-genome sequencing (WGS) offers ideal resolution to describe population dynamics and to predict and infer transmission of antimicrobial resistance, and can enhance infection control through linkage with epidemiological data. We used WGS, in conjunction with linked epidemiological and phenotypic data, to describe the gonococcal population in 20 European countries. We aimed to detail changes in phenotypic antimicrobial resistance levels (and the reasons for these changes) and strain distribution (with a focus on antimicrobial resistance strains in risk groups), and to predict antimicrobial resistance from WGS data.

**Methods:**

We carried out an observational study, in which we sequenced isolates taken from patients with gonorrhoea from the European Gonococcal Antimicrobial Surveillance Programme in 20 countries from September to November, 2013. We also developed a web platform that we used for automated antimicrobial resistance prediction, molecular typing (*N gonorrhoeae* multi-antigen sequence typing [NG-MAST] and multilocus sequence typing), and phylogenetic clustering in conjunction with epidemiological and phenotypic data.

**Findings:**

The multidrug-resistant NG-MAST genogroup G1407 was predominant and accounted for the most cephalosporin resistance, but the prevalence of this genogroup decreased from 248 (23%) of 1066 isolates in a previous study from 2009–10 to 174 (17%) of 1054 isolates in this survey in 2013. This genogroup previously showed an association with men who have sex with men, but changed to an association with heterosexual people (odds ratio=4·29). WGS provided substantially improved resolution and accuracy over NG-MAST and multilocus sequence typing, predicted antimicrobial resistance relatively well, and identified discrepant isolates, mixed infections or contaminants, and multidrug-resistant clades linked to risk groups.

**Interpretation:**

To our knowledge, we provide the first use of joint analysis of WGS and epidemiological data in an international programme for regional surveillance of sexually transmitted infections. WGS provided enhanced understanding of the distribution of antimicrobial resistance clones, including replacement with clones that were more susceptible to antimicrobials, in several risk groups nationally and regionally. We provide a framework for genomic surveillance of gonococci through standardised sampling, use of WGS, and a shared information architecture for interpretation and dissemination by use of open access software.

**Funding:**

The European Centre for Disease Prevention and Control, The Centre for Genomic Pathogen Surveillance, Örebro University Hospital, and Wellcome.

## Introduction

Gonorrhoea and antimicrobial resistance in *Neisseria gonorrhoeae* are public health concerns globally. Clinical resistance to the last options of the first-line therapies of gonorrhoea, the extended-spectrum cephalosporins (namely, cefixime and ceftriaxone) and azithromycin, has emerged.^1^, ^2^, ^3^, ^4^, ^5^ This worrying situation, along with the potentially severe complications of untreated gonorrhoea, mean that gonorrhoea is a global public health problem.^1^, ^2^, ^5^, ^6^

The European Gonococcal Antimicrobial Surveillance Programme (Euro-GASP) monitors antimicrobial resistance in gonococci in the European Union/European Economic Area (EU/EEA; appendix 1).^3^, ^4^, ^6^, ^7^ Euro-GASP also performs molecular typing of gonococcal isolates, particularly to describe the gonococcal population in the EU/EEA, transmission of antimicrobial resistant strains, and associations with epidemiological characteristics of the patients. In a previous Euro-GASP molecular typing study,^8^ 1066 isolates collected in 2009–10 were typed with *N gonorrhoeae* multi-antigen sequence typing (NG-MAST). The key findings included a high prevalence of the multidrug-resistant NG-MAST genogroup 1407 (G1407) across the EU/EEA. G1407 accounted for nearly all resistance to cefixime, the first-line antimicrobial for monotherapy in the EU/EEA at that time, and showed decreased susceptibility or resistance (or both) to ceftriaxone, azithromycin, and ciprofloxacin.^8^

**Research in context**

**Evidence before this study**

We searched PubMed using the terms “*Neisseria gonorrhoeae”* OR “gonorrhoea” with “sequencing” OR “molecular epidemiology” for papers published in English between Jan 1, 2000, and Dec 5, 2017. Previously, whole genome sequencing (WGS) of *N gonorrhoeae* was mainly used to investigate molecular epidemiology at a national or local level in the USA (national: isolates with reduced cephalosporin susceptibility and cephalosporin-susceptible isolates [236 isolates] and isolates resistant to cephalosporins, azithromycin, and ciprofloxacin [1102 isolates]), Canada (national: azithromycin-resistant isolates [246 isolates] and isolates with decreased cephalosporin susceptibility [169 isolates]), the UK (Brighton: 1407 isolates), and Brazil (Rio de Janeiro: 116 isolates), and have focused on a few isolates that were selected because of their antimicrobial resistance, for examining single strains or local outbreaks (including determining transmission). To our knowledge, except for one study that examined a low number of selected azithromycin-resistant isolates (75 isolates) from 17 European countries, there have been no regional studies that used WGS of *N gonorrhoeae*.

**Added value of this study**

To our knowledge, we report the first use of WGS in conjunction with relevant epidemiological and phenotypic data in an international programme for regional surveillance of sexually transmitted infections and we improve the understanding of the distribution of resistant clones in different risk groups nationally and regionally. For simplified, appropriate analysis in gonococcal genomic projects, a publicly available and user-friendly web application was adapted and optimised for *N gonorrhoeae* and used for all analyses and dissemination of results to participating countries. We used WGS to investigate 1054 Euro-GASP isolates (consecutive susceptible and resistant isolates) that were collected in 20 European countries in 2013 and we describe the genomic baseline of the European gonococcal population, identify antimicrobial resistant clades and clones and their associations with patient metadata, predict antimicrobial resistance, and compare our results with a European molecular survey from 2009–10. We show that WGS produces a significantly higher and more accurate resolution of gonococcal isolates than do other molecular epidemiological typing methods, such as *N gonorrhoeae* multi-antigen sequence typing and multilocus sequence typing, such that discrepant isolates, mixed infections, and contaminants can be identified in surveillance programmes by use of our pipeline, and that antimicrobial resistance can be accurately predicted in most cases.

**Implications of all the available evidence**

Use of WGS in conjunction with relevant epidemiological and phenotypic data in *N gonorrhoeae* surveillance programmes provides enhanced understanding of the distribution of antimicrobial resistance clades and clones in different risk groups nationally and regionally. Current molecular methods, such as *N gonorrhoeae* multi-antigen sequence typing and multilocus sequence typing, do not provide the resolution nor accuracy necessary for molecular epidemiology, and their use should be discouraged. WGS can additionally identify discrepant isolates, mixed infections, and contaminants, and predict antimicrobial resistance. The provision of data through a simple-to-use web application that enables ongoing addition and analysis of new genomic data with rapid phylogenetic analysis and antimicrobial resistance prediction within an epidemiological context enables the ongoing national and regional surveillance of *N gonorrhoeae* with WGS.

Since 2010, the patterns of antimicrobial resistance in gonococci have changed in the EU/EEA. In 2012, combination therapy (ceftriaxone plus azithromycin), which was already being used in several countries, was officially recommended for all the European countries.^9^, ^10^ Since then, cefixime resistance has decreased and azithromycin resistance has increased.^4^, ^9^ It is crucial to understand whether these changes to resistance are due to the introduction of combination therapy,^10^ or changes to sexual behaviour in sexual networks or the gonococcal population, other reasons, or a combination of these factors. Linkage of molecular typing data to antimicrobial resistance and epidemiological data can elucidate changes to strain distributions overall and among risk groups.

Whole-genome sequencing (WGS) allows finer resolution and improved accuracy compared with typing schemes that are based on a small number of genetic loci. With WGS, the true baseline of genomic heterogeneity of the gonococcal population and the spread of antimicrobial resistance among gonococcal strains in the EU/EEA can be described (for instance, to inform outbreak investigations). We can elucidate the associations of specific gonococcal strains or clades with antimicrobial resistance profiles (including genetic determinants of antimicrobial resistance) for therapeutic antimicrobials and for risk groups, nationally and at the EU/EEA level, to guide public health interventions. WGS has not previously been used within a structured regional programme for public health surveillance of sexually transmitted infections for several reasons. These reasons include cost, scale, or an absence of automated systems and intuitive interpretation methods linking genomics to antimicrobial resistance and epidemiological data and providing data in formats that can inform the public health response, or a combination of these factors.

We aimed to use WGS of gonococcal isolates from 20 EU/EEA countries in conjunction with epidemiological and phenotypic data to describe the genomic baseline of the European gonococcal population, identify antimicrobial resistance clades and clones and their associations with patient metadata, predict antimicrobial resistance, and compare our results to the Euro-GASP 2009–10 NG-MAST survey.^8^ For simplified, appropriate analysis in gonococcal genomic projects, we also aimed to make data available through development of a publicly available and user-friendly web application for gonococci, for analysis and dissemination of results to participating countries.

## Methods

### Euro-GASP sampling

From Sept 1 to Nov 30, 2013, 1218 isolates that were linked to epidemiological information were collected from clinics and hospitals in 21 Euro-GASP countries. In accordance with the Euro-GASP protocols,^3^, ^4^, ^7^, ^9^ laboratories collect only one isolate per patient from those who were infected multiple times within a 4-week period or at several anatomical sites, to represent different gonorrhoea episodes. For this study, each country was asked to, where possible, contribute 50 isolates from September to November, 2013. In Spain, the Netherlands, and the UK, where 50 isolates represented fewer than 10% of the total gonorrhoea cases, 100 isolates were requested. Furthermore, some countries with fewer than 50 isolates from September to November, 2013 (Cyprus, Iceland, and Malta), were asked to add previous isolates, giving an extended study duration (appendix 1).^3^, ^4^, ^7^, ^9^

After decentralised (12 countries) or centralised (eight countries) antimicrobial susceptibility testing, all countries participating in Euro-GASP reported their antimicrobial susceptibility data through The European Surveillance System (European Centre for Disease Prevention and Control, Stockholm, Sweden). In addition to the antimicrobial susceptibility data, countries reported epidemiological data that were linked to the gonorrhoea cases through The European Surveillance System.^3^, ^4^, ^7^ Epidemiological data collected with each isolate, where possible, included the date that the clinical specimen was obtained, the anatomical site that the specimen was isolated from, sex, age, sexual orientation, previous gonorrhoea diagnosis, other sexually transmitted infections diagnosed during the current gonorrhoea infection, place of residence, type of clinic that the patient visited, HIV status, and probable country of infection.

All gonococcal isolates were cultured as part of routine diagnostics, and isolates or data were submitted to the Euro-GASP surveillance study with no information that would enable patient identification; ethical approval was, therefore, not required. We tested viable isolates for which country representatives had permitted molecular typing with WGS. Antimicrobial susceptibility testing was done to determine the minimum inhibitory concentration (MIC) with Etests (bioMérieux; Marcy-l'Étoile, France) for cefixime and ceftriaxone, and an agar dilution breakpoint method or Etests for ciprofloxacin, azithromycin, and spectinomycin. SIR (susceptible, intermediate, resistant) breakpoints from the European Committee on Antimicrobial Susceptibility Testing were applied.

### WGS procedures and analyses

We extracted DNA from a single colony of each isolate with a Wizard Genomic DNA Purification kit (Promega; Madison, WI, USA) or a QIAsymphony instrument (Qiagen; Hilden, Germany), and whole-genome sequenced purified DNA extracts, as described (appendix 1).^11^

We derived multilocus sequence typing (MLST) sequence types, NG-MAST sequence types, and NG-MAST genogroups in silico from the WGS data. Known antimicrobial resistance determinants (appendix 2) were searched against the sequence assemblies of the isolates by use of BLASTn under default parameters. Antimicrobial resistance determinants were grouped into sets of one or more that collaboratively provide antimicrobial resistance. Complete sets indicate intermediate or full resistance predicted to the specified antimicrobial drug; incomplete sets may confer intermediate or no resistance (appendix 2).

We examined the associations between NG-MAST genogroups, antimicrobial resistance, and patient characteristics (sex, age, and sexual orientation; appendices 1 and 2).^12^, ^13^, ^14^, ^15^ The statistical analyses are detailed in appendix 1. Changes in phenotype distribution were assessed with TreeBreaker (appendix 1).^16^

### Role of the funding source

The funders of the study had no role in study design, data collection, data analysis, data interpretation, or writing of the report of this study. The corresponding authors had full access to all the study data and had final responsibility for the decision to submit for publication.

## Results

Quality-checked WGS data linked to antimicrobial resistance profiles and epidemiological data were obtained for 1054 (87%) of 1218 isolates from 20 countries (no isolates available from Ireland). Sex of the patient was reported for 1045 (99%) of 1054 isolates and age of the patient was reported for 1023 (97%) isolates. Among the 1045 patients with reported sex, 884 (85%) were male and 161 (15%) were female. The median age was 31 years for men and 24 years for women, with a peak in the 25–30 age group for men (185 [21%] of 861 isolates with reported patient age and sex) and in the 20–29 age group in women (50 [31%] of 160 isolates with reported patient age and sex). Sexual orientation of the patient was reported for 499 (47%) of isolates, of which 294 (59%) were from a patient with a reported heterosexual orientation. Of the 414 (47%) men who reported their sexual orientation, 206 (50%) reported being men who have sex with men (MSM).

Antimicrobial resistance in the 1054 isolates is summarised in table 1. 562 (53%) isolates showed phenotypic resistance to ciprofloxacin, 71 (7%) to azithromycin, 51 (5%) to cefixime, five (<1%) to ceftriaxone, and none to spectinomycin (before re-testing of discrepant isolates identified through WGS). Resistance to ciprofloxacin across countries ranged from 28 (26%) of 106 isolates in the UK to seven (88%) of eight isolates in Cyprus, and resistance to azithromycin from 14 (29%) of 48 isolates in Greece to none in nine countries. Cefixime-resistant isolates were identified in 12 (60%) countries, and resistance varied from 11 (23%) of 48 isolates in Greece to none in nine countries. Five (<1%) ceftriaxone-resistant isolates were identified in Spain.

**Table 1:** Phenotypic antimicrobial resistance of isolates from each country (before retesting of discrepant isolates [phenotype *v*sgenotype])

	**Number of isolates**	Ciprofloxacin resistance*	Azithromycin resistance*	Cefixime resistance*	Ceftriaxone resistance*	Spectinomycin resistance*
Austria	54	42 (78%)	1 (2%)	0	0	0
Belgium	55	29 (53%)	0	2 (4%)	0	0
Cyprus	8	7 (88%)	2 (25%)	0	0	0
Denmark	55	29 (53%)	4 (7%)	9 (16%)	0	0
France	57	30 (53%)	0	3 (5%)	0	0
Germany	47	23 (49%)	1 (2%)	1 (2%)	0	0
Greece	48	34 (71%)	14 (29%)	11 (23%)	0	0
Hungary	48	35 (73%)	0	2 (4%)	0	0
Iceland	5	2 (40%)	0	0	0	0
Italy	26	13 (50%)	0	0	0	0
Latvia	38	10 (26%)	6 (16%)	1 (3%)	0	0
Malta	20	8 (40%)	0	0	0	0
Netherlands	66	24 (36%)	1 (2%)	0	0	0
Norway	55	44 (80%)	6 (11%)	1 (2%)	0	0
Portugal	108	50 (46%)	21 (19%)	0	0	0
Slovakia	38	18 (47%)	0	1 (3%)	0	0
Slovenia	54	33 (61%)	0	0	0	0
Spain	116	75 (65%)	10 (9%)	18 (16%)	5 (4%)†	0
Sweden	50	28 (56%)	5 (10%)	0	0	0
UK	106	28 (26%)	0	2 (2%)	0	0
All	1054	562 (53%)	71 (7%)	51 (5%)	5 (<1%)†	0

Data are number of isolates, with % where relevant.

* Resistance breakpoints determined by EUCAST.

† All were susceptible in centralised retesting, which was done because of discrepancies with the whole-genome sequencing data.

MLST and NG-MAST sequence types and genogroups, including associations with patient epidemiological characteristics and antimicrobial resistance, are shown in table 2 and the appendix 2. Isolates were assigned to 103 MLST sequence types and 377 NG-MAST sequence types (160 NG-MAST genogroups), of which 23 MLST sequence types and 17 NG-MAST sequence types included ten or more isolates. 35 (34%) of 103 MLST sequence types, 249 (66%) of 377 NG-MAST sequence types, and 84 (53%) of 160 NG-MAST genogroups contained only one isolate. As in 2009–10,^8^ NG-MAST G1407 (n=174 isolates) was the predominant genogroup and was significantly associated with resistance to azithromycin, ciprofloxacin, and cefixime (p<0·0001 with χ^2^) and an increased MIC of ceftriaxone (p<0·0001 with logistic regression; appendix 2). However, the prevalence of G1407 decreased significantly from 2009–10 to 2013, from 248 (23%) of 1066 isolates^8^ to 174 (17%) of 1054 isolates (p<0·0001 in a *Z*-test). Furthermore, in 2013, G1407 was significantly associated with patients who were older (at least 45 years; odds ratio [OR] 1·01 [95% CI 1·00–1·03]; p=0·0463) and heterosexual (4·29 [2·12–8·67]; p<0·0001; appendix 2), whereas, in 2009–10, G1407 was associated with MSM.^8^ However, G1407 was also associated with missing sexual orientation metadata (1·86 [1·32–2·61]; p=0·0004 with χ^2^), which might confound these results if absence of data was not random. G2400 and G4995, associated with MSM, and G51, associated with young (age 15–24 years) heterosexual people, were more prevalent in 2013 than in 2009–10 (appendix 2).^8^

**Table 2:** Distribution of NG-MAST sequence types and genogroups and MLST sequence types in *Neisseria gonorrhoeae* isolates from the 20 European countries in Euro-GASP, 2013

	**Number of isolates**	**Number of whole genome sequences**	**Number of NG-MAST sequence types**	**Most common NG-MAST sequence types (number of isolates)**	**Number of NG-MAST genogroups**	**Most common genogroup (number of isolates)**	**Number of MLST sequence types**	**Most common MLST sequence types (number of isolates)**
Austria	55	54	24	3785 (9)	20	G3785 (9)	17	1901 (12)
Belgium	55	55	24	1407 (9)	23	G1407, G2992 (9 each)	20	1901 (11)
Cyprus	9	8	4	1407 (3)	1	G1407 (6)	3	1901 (6)
Denmark	56	55	28	1993 (11)	23	G1407 (12)	20	1901 (14)
France	58	57	40	645 (5)	28	G645, G2992 (6 each)	24	1901, 7363 (8 each)
Germany	50	47	30	4995 (4)	16	G1407 (10)	17	1901 (11)
Greece	50	48	20	3128 (9)	16	G1407 (12)	12	1901 (25)
Hungary	50	48	20	1407 (10)	11	G1407 (20)	10	1901 (21)
Iceland	5	5	5	1034, 2400, 9541, 10 640, 11 080 (1 each)	5	G21, G995, G2400, G9541, G11080 (1 each)	5	1579, 7363, 8156, 9363, 11 979 (1 each)
Italy	50	26	13	2992 (8)	10	G2992 (9)	12	9363 (5)
Latvia	38	38	15	5 (14)	10	G21 (18)	7	1579 (19)
Malta	31	20	10	2992 (7)	8	G2992 (10)	9	11 428 (6)
Netherlands	88	66	38	2992 (9)	22	G2992 (12)	21	7363 (12)
Norway	55	55	41	1407 (5)	32	G1407 (10)	23	1901 (16)
Portugal	112	108	54	1407 (17)	35	G1407 (26)	32	1901 (22)
Slovakia	56	38	19	1407, 10 800, 11 042 (5)	12	G51 (12)	8	1901 (13)
Slovenia	73	54	26	21 (7)	17	G21 (10)	15	1579, 1588 (9 each)
Spain	119	116	64	1407 (11)	35	G1407 (23)	30	1901 (30)
Sweden	50	50	31	5445 (5)	24	G1407 (8)	18	1901 (14)
UK	110	106	52	2992 (12)	38	G51 (14)	35	9363 (14)
All	1170	1054	377	1407 (78)	160	G1407 (174)	103	1901 (166)

NG-MAST=*Neisseria gonorrhoeae* multi-antigen sequence typing. MLST=multilocus sequence typing.

Six isolates were contaminated with a substantial amount of non-gonococcal DNA (>100 kb of DNA from *Mycoplasma* spp). 23 isolates contained mixed gonococcal sequences, indicating mixed infections, contamination, or both.

Genomic antimicrobial resistance determinants,^1^ which were identified by Whole Genome Sequence Analysis (WGSA) are detailed in appendix 2. On the basis of the genomic results, 34 discrepant isolates were identified and re-phenotyped. 17 isolates that were originally reported as ciprofloxacin-resistant, but had no known ciprofloxacin resistance mutations, re-tested as susceptible, and four isolates reported as susceptible but that contained a *gyrA* Ser91Phe substitution, which is known to confer ciprofloxacin resistance, re-tested as resistant. Two additional isolates with a *gyrA* Ser91Phe substitution had ciprofloxacin MICs of less than 0·06 mg/L even after re-testing. PCR showed that neither of the phenotypically tested isolates had a *gyrA* Ser91Phe substitution, which suggested a sample mix-up. Ten isolates reported to be cefixime-resistant re-tested as susceptible, and one isolate that was reported to be cefixime-susceptible was resistant in re-testing. Although all five ceftriaxone-resistant isolates from Spain possessed the mosaic *penA*XXXIV allele that is associated with decreased susceptibility or resistance to extended-spectrum cephalosporins, they did not have a unique genotype to explain the resistance, and did not cluster on the WGSA tree. All five isolates were therefore re-tested and found to be susceptible to ceftriaxone. The MICs of all isolates with discrepant results regarding susceptibility to extended-spectrum cephalosporins were close to the resistance breakpoint (>0·125 mg/L) and were initially tested via decentralised testing with agar dilution, whereas centralised re-testing was done with Etests.

**Figure 1:**
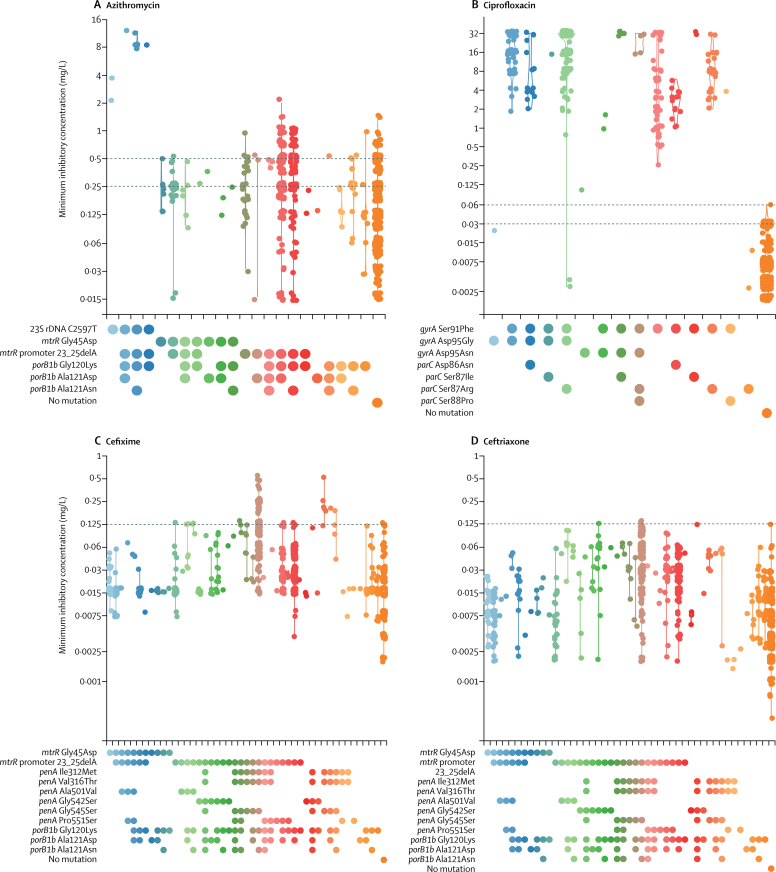
Violin plots of observed minimum inhibitory concentrations for combinations of known genotypic antimicrobial resistance determinants or without any of these mutations Minimum inhibitory concentrations are on the *y*-axis. Combinations of resistance determinants are on the *x*-axis; different colours indicate different mutation combinations. Data were recorded after re-testing. Dashed horizontal lines indicate breakpoints from the European Committee on Antimicrobial Susceptibility Testing.

Figure 1 shows the MICs for isolates (after re-testing) that showed combinations of the antimicrobial resistance determinants in appendix 2. Ciprofloxacin resistance (in 549 isolates) was only found in isolates containing a *gyrA* Ser91Phe substitution, except for one isolate from Spain (MIC=0·094 mg/L), shown in light green. This isolate was the only one that contained a *gyrA* Asp95Asn substitution without also containing the Ser91Phe substitution, which indicates that, in rare isolates, *gyrA* Asp95Asn alone might cause low-level ciprofloxacin resistance. 71 isolates were azithromycin-resistant. No isolates had high-level azithromycin resistance (MIC≥256 mg/L) or had the associated 23S rDNA 2045A→G (2059A→G *Escherichia coli* numbering) mutation. All but one isolate with azithromycin MICs of at least 2 mg/L (11 isolates) had the 2597C→T (2611C→T *E coli* numbering) 23S rDNA mutation, which was not seen in isolates with MICs of less than 2 mg/L. The remaining isolate (azithromycin MIC=2 mg/L) contained the *mtrR* promoter 23_25delA deletion, which is associated with increased azithromycin MICs. Decreased susceptibility and resistance to cefixime was particularly associated with *penA*X and *penA*XXXIV alleles (which contain the mosaic *penA* allele indicator substitutions Ile312Met, Val316Thr, and Gly545Ser). All isolates with a cefixime MIC of more than 0·125 mg/L had a mosaic *penA*X (six isolates), *penA*XXXIV (35 isolates), or a novel allele differing from *penA*XXXIV by a single aminoacid substitution (one isolate). However, 158 isolates with mosaic *penA* alleles had a cefixime MIC of less than or equal to 0·125 mg/L, including one *penA*X, 153 *penA*XXXIV, three *penA*LXXII, and one novel allele. The latter two alleles differ from *penA*XXXIV by a single aminoacid substitution.

Even after re-testing, the antimicrobial MIC values of some genotypes clearly varied between participating countries, despite isolates being intermingled in the phylogeny (appendix 1). Countries with decentralised testing often reported higher MICs for cefixime, whereas ceftriaxone MICs for Portuguese isolates were significantly lower than those for all other countries (p<0·0001 with a Kolmogorov-Smirnov test).

**Figure 2:**
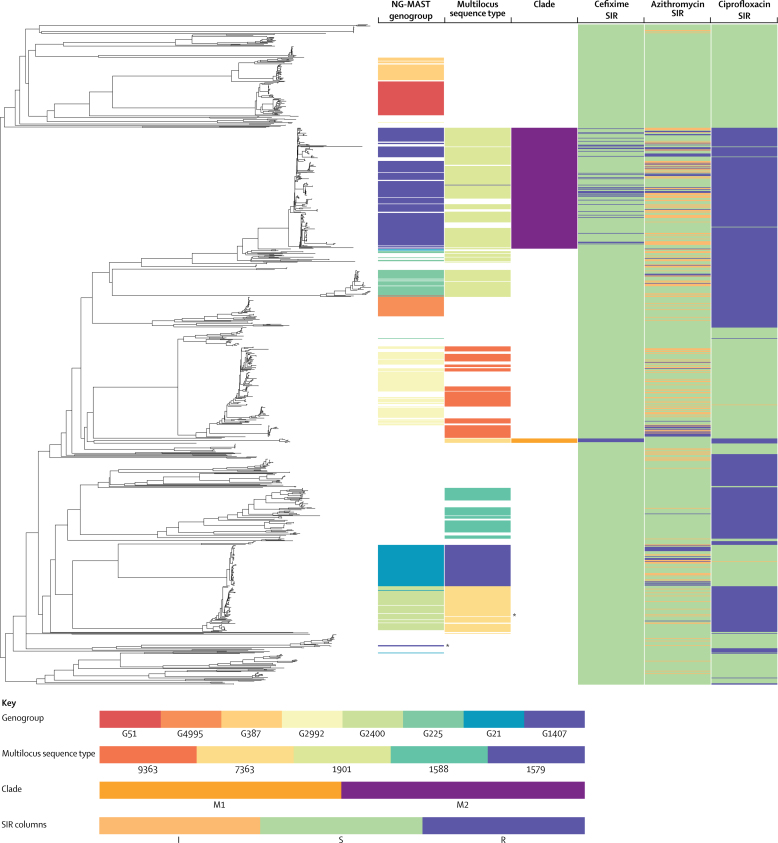
Comparison of whole-genome sequencing, NG-MAST genogrouping, and multilocus sequence typing Data are the phylogenetic tree from the whole-genome sequence analysis from Euro-GASP, 2013. Columns indicate the location of isolates in the eight most prevalent *Neisseria gonorrhoeae* multiantigen sequence typing genogroups, the five most prevalent multilocus sequence types, whole-genome sequence clades M1 and M2 (defined from the phylogenetic tree), and the SIR data for cefixime, azithromycin, and ciprofloxacin. NG-MAST=*Neisseria gonorrhoeae* multi-antigen sequence typing. SIR=susceptible, intermediate, resistant. *Secondary clades of NG-MAST genogroup 1407 and multilocus sequence type 7363 isolates. Figure produced with Phandango.^17^

WGSA identified 41 639 variable sites between 921 genotypes in the core genomes of the sequenced isolates. The whole-genome phylogeny (figure 2) revealed a population structure that was generally concordant with MLST (consistency index 0·843, retention index 0·974) and NG-MAST (0·922, 0·944). These concordances are artificially inflated by the high number of uninformative singleton MLST (35 of 103 MLST sequence types) and NG-MAST (249 of 377 NG-MAST sequence types) sequence types identified, which provide no information on isolate relationships and are concordant with any phylogeny. For both MLST and NG-MAST, after exclusion of mixed samples, incorrect clusterings that were identified by use of the WGS tree were explained by sequence type convergences or reversions that were primarily caused by homologous recombination of typing alleles. Clustering of NG-MAST into genogroups compounded this effect, leading to increased levels of genogroup homoplasy on the tree (0·795, 0·941).

The shortcomings of MLST and NG-MAST can be illustrated, for example, by isolates possessing mosaic *penA* alleles, which formed two discrete clades in the WGS phylogeny (figure 2). One clade contained *penA*X (clade M1) and the other contained *penA*XXXIV (clade M2). Neither MLST nor NG-MAST accurately associated isolates with these two clinically important clades. M1 comprised seven isolates from six countries, which had been assigned to five NG-MAST genogroups and a single MLST (sequence type 7363). Although all M1 isolates shared a single MLST, sequence type 7363 also included a larger group of phylogenetically distant isolates that did not have *penA*X alleles and showed lower cefixime MICs than the isolates in the M1 clade (M1: mean=0·234, SD=0·123 [seven M1 clade isolates]; 71 non-M1 sequence type 7363: 0·040, 0·022). M2 comprised 193 isolates in 11 NG-MAST genogroups (58 NG-MAST sequence types) dominated by G1407 (172 isolates) and eight MLST sequence types, dominated by sequence type 1901 (166 isolates). Neither NG-MAST G1407 nor MLST sequence type 1901 was restricted to this clade. MLST sequence type 1901 included a further 58 related isolates that did not have a mosaic *penA*, whereas G1407 included two phylogenetically distant isolates that seemed to have undergone recent recombination events at both NG-MAST alleles (appendix 1), rendering them indistinguishable with NG-MAST. Neither of these isolates possessed a mosaic *penA* allele, and both had cefixime MICs of 0·016 mg/L compared with a mean of 0·124 mg/L (SD 0·105) for all other G1407 isolates.

An alternative to typing methods such as NG-MAST or MLST is to identify important clades by examination of genotypic or phenotypic markers, such as mosaic *penA* alleles, SIR data, or patient demographic data. This strategy allows more accurate monitoring of clades or subclades of the gonococcal population that pose the biggest threat to public health and it can potentially aid decisions on treatment and interventions. The TreeBreaker algorithm^16^ divided the branches leading to clades M1 and M2 with only cefixime SIR data (appendix 1). Similarly, results for azithromycin and ciprofloxacin SIR (appendix 1) identified clades of importance for these drugs. Four clades were associated with an increased incidence of azithromycin-resistant or intermediate-resistant isolates, whereas five were associated with ciprofloxacin resistance, consistent with several gains of the *gyrA* Ser91Phe substitution. By use of reported patient sexual orientation to identify clades (appendix 1), five clades were identified that were associated with heterosexual people, including a basal split in the tree between an MSM-associated clade and a heterosexual-associated clade, which later transmitted back into an MSM population.

**Figure 3:**
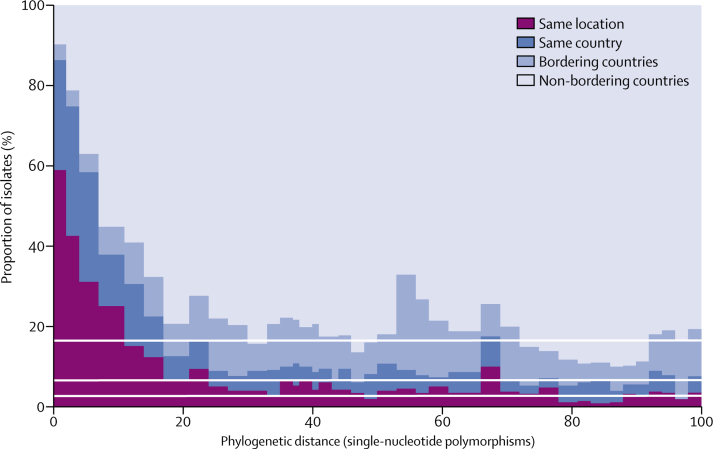
Histogram of pairwise phylogenetic distance of isolates on the Whole Genome Sequence Analysis tree Data are split into four geographical categories. White lines indicate the splits of geographical categories for all isolates. Phylogenetic distance data are presented up to 100 single-nucleotide polymorphisms.

The superior resolution of WGS versus NG-MAST and MLST, and linking to epidemiological data, has the potential to allow early identification of novel outbreak or high-risk clones (eg, as defined by antimicrobial resistance). Analysis of pairwise geographical and cophenetic distances (distances between pairs of isolates on the tree; figure 3) showed that geographical mixing occurred very rapidly. The geographical separation of two isolates greater than 30 single nucleotide polymorphisms apart was essentially random, whereas about 80% of isolates separated by one single nucleotide polymorphism (or not separated) were from the same country (and about 60% from the same city or region), despite only about 7% of isolate pairs being from the same country.

**Figure 4:**
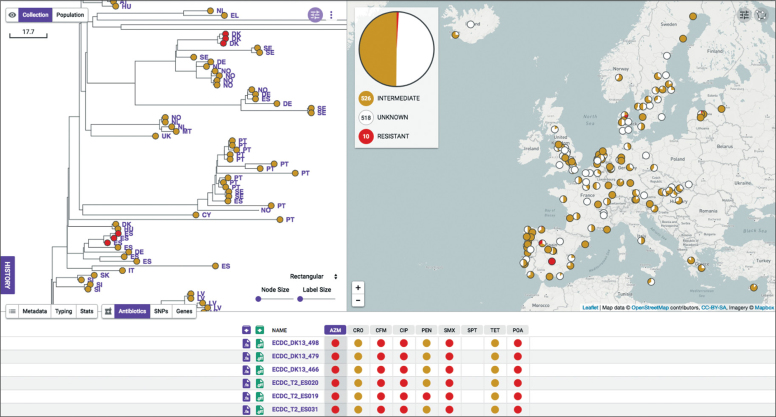
Whole Genome Sequence Analysis screenshot of genomic epidemiology of two minor clusters of multi-drug resistant *Neisseria gonorrhoeae* Data in the top left box are the phylogenetic reconstruction of the relationships of isolates in part of the Euro-GASP tree, generated by the Whole Genome Sequence Analysis web application. Red circles are isolates belonging to the two clusters with predicted resistance to azithromycin, based on the presence of known genetic determinants. Branch two-letter labels represent the country of origin of the isolates. The scale bar relates to horizontal branch lengths and indicates the number of single nucleotide polymorphisms that are proposed to have occurred on the branches. Data in the top right panel are the geographical distribution of isolates in the collection. The red pie charts at each location indicate predicted resistance to azithromycin. The larger pie chart is the total predicted azithromycin resistance in the entire collection. Data in the lower panel are the predicted resistance profiles of the six isolates that are highlighted in red in the top left panel. Red circles indicate predicted resistance and orange circles indicate where known determinants of decreased susceptibility are present, although these determinants do not necessarily lead to resistance.

The development of resistance to dual gonorrhoea therapy (ceftriaxone plus azithromycin) is of particular concern. WGSA allowed rapid identification of two clades (figure 4), each comprising three closely related isolates (two to five single nucleotide polymorphisms distant), that contained a mosaic *penA*XXXIV that conferred decreased susceptibility to extended-spectrum cephalosporins, a 23S rRNA 2597C→T mutation that conferred azithromycin resistance, and *gyrA* Ser91Phe substitution that conferred ciprofloxacin resistance. The MICs of all six isolates were 8–16 mg/L for azithromycin and more than 32 mg/L for ciprofloxacin. The cefixime MICs for four isolates were at least 0·125 mg/L, but all were susceptible to ceftriaxone. The epidemiological characteristics of the two clades were strikingly similar. In both cases, the three isolates were from the same country (Spain and Denmark), with a single isolate from a heterosexual man isolated 1 month before the two other isolates, which were from heterosexual men and women and were isolated on a single day in the same location. These characteristics clearly support the genomic evidence that suggest that these isolates are epidemiologically linked.

## Discussion

We report the findings of the first Euro-GASP WGS-based structured gonococcal survey in the EU/EEA. The multidrug-resistant NG-MAST genogroup G1407, which has been previously described in the EU/EEA,^8^, ^18^ USA,^19^, ^20^ and Canada^21^, ^22^ is decreasing in prevalence because of replacement by more cephalosporin-susceptible geno-groups. An association between G1407 and heterosexuals was also indicated in this 2013 survey, in contrast with the association with MSM seen in 2009–10.^8^ This decrease in G1407 prevalence and changes in its epidemiological features could be due to many factors. Such possible factors include the increased use of more sensitive molecular diagnostics and increased testing of extra-genital sites (particularly the pharynx) among MSM, which have contributed to improved detection of the G1407 reservoir. This increase in detection has enabled appropriate and effective treatment with combination therapy (ceftriaxone plus azithromycin).^10^ By contrast, the previous first-line therapy (cefixime monotherapy) was not effective against cefixime-resistant G1407 infections, particularly in pharyngeal infections. G1407 appears to have been replaced by cefixime-susceptible genogroups that might have a higher fitness. Thus, four of the most prevalent genogroups in 2013 (G21, G51, G2400, G4995) were not common in 2009–10 (appendix 1), illustrating the instability of the gonococcal population in the EU/EEA, and why constant monitoring of the population is essential. These four genogroups were associated with known risk groups (MSM and young heterosexuals), illustrating that new clones can expand rapidly in these populations.

We showed in regional surveillance programmes of sexually transmitted infections internationally that WGS has many benefits over NG-MAST and MLST, which are suboptimal for molecular epidemiology. For example, WGS identifies mixed infections and contaminants in samples, accurately predicts antimicrobial resistance in many cases, highlights inconsistencies between genotypic and phenotypic antimicrobial resistance, and provides greatly improved epidemiological accuracy and resolution. WGS also allows identification of important gonococcal clades or clones on the basis of informative genomic markers, rather than typing alleles with no relevance to clinically important phenotypes. For example, the NG-MAST genogroup G1407 excludes isolates in the same WGS clade with identical antimicrobial resistance phenotypes and that contain the mosaic *penA*XXXIV allele, while simultaneously including unrelated isolates that neither contain *penA*XXXIV nor show increased extended-spectrum cephalosporin MIC. Although NG-MAST genogroups fitted the WGS phylogeny relatively well, these and other inaccuracies (figure 2) highlight the flaws of MLST and NG-MAST, which simultaneously split clinically important, phylogenetically related isolates, and include phylogenetically unrelated, less relevant isolates. Such situations result in reduced power for statistical and epidemiological analyses (appendix 2). Furthermore, because of the NG-MAST genogrouping methodology, two of the most prevalent genogroups (G25 and G2992) in the 2009–10 Euro-GASP survey^8^ became merged in our analysis, making comparison between the two surveys difficult. Over time, such problems will worsen as variation in the NG-MAST and MLST alleles continues to accrue, and will affect our ability to track emerging clones with these methods.

We also adapted, optimised, and used *N gonorrhoeae* WGSA as a mechanism for user-friendly, publicly accessible, WGS-based molecular epidemiology to allow rapid analysis and visualisation of genomic phylogenetic relationships and the results of in-silico molecular typing with geographical and patient metadata. Nevertheless, to fully benefit from ongoing genome-based molecular epidemiological surveillance by use of WGSA, reporting of metadata in Euro-GASP and other programmes or projects needs to improve. WGSA also predicts antimicrobial resistance from genomic markers. Although genotypic prediction of antimicrobial resistance shows great promise, it will never completely replace phenotypic antimicrobial resistance testing because novel and unknown antimicrobial resistance determinants cannot be identified, and the correlations between many antimicrobial resistance determinants and the MICs of antimicrobials are currently suboptimal. Genotypic predictions will, however, supplement phenotypic testing by highlighting discrepancies to inform re-testing and strengthen the external quality assessment programme of Euro-GASP. We showed that WGS highlighted several cases where the antimicrobial resistance phenotype was incorrect. Centralised re-phenotyping supported the genotypic prediction in most cases. Furthermore, the phenotypic antimicrobial resistance results for phylogenetically closely related isolates varied between participating countries, particularly where testing was decentralised. Concordantly, Eyre and colleagues^23^ showed that gonococcal MICs from a global convenience collection could only be accurately predicted from the genotype if the country of testing was included in the model. Such inconsistencies might negatively affect the clinical relevance of Euro-GASP surveillance, breakpoints from the European Committee on Antimicrobial Susceptibility Testing, our ability to monitor the distribution and spread of antimicrobial resistance, and our ability to improve the accuracy of genomic antimicrobial resistance prediction. Despite the small number of discrepant isolates, this highlights the clinical importance of standardised and quality-assured phenotypic testing and WGS analysis in future Euro-GASP surveys.

As previously described,^4^, ^7^ the main limitations of Euro-GASP include the low number and possibly suboptimal representativeness of isolates from some countries, lower number of isolates from women, and low level of reporting of some key epidemiological variables, such as sexual orientation. However, the representativeness of Euro-GASP was evaluated in detail in 2017. This study^24^ showed that the overall prevalence of antimicrobial resistance reported by Euro-GASP appropriately reflects the antimicrobial resistance situation in EU/EEA and weighting of the data did not provide very different overall antimicrobial resistance estimates. Nevertheless, improving the representativeness of Euro-GASP remains crucial and this work is part of the ongoing Euro-GASP work programme.

Genomic epidemiology of gonococci using WGSA also allows rapid, interactive, and nearly real-time tracking of high-risk clones and identification of outbreaks. These clones and outbreaks can be visualised with associated epidemiological characteristics and antimicrobial resistance data for clinical and public health purposes, such as to guide enhanced testing and screening, tailored treatment, or interventions for infection control in risk groups at national or international levels. More broadly, lineages associated with antimicrobial resistance or patient demographics can be identified with methods such as TreeBreaker,^16^ which identify changing distributions of variables across the phylogenetic tree. This ability to automatically and rapidly identify changes in the demographics of the host population illustrates the clinical and epidemiological potential of genomic epidemiology to monitor high-risk transmission networks. On a smaller scale, interactive visualisation in WGSA allowed us to identify two short transmission chains within individual participating countries that showed resistance to azithromycin and ciprofloxacin and decreased susceptibility to extended-spectrum cephalosporins. It is important to identify such lineages quickly to allow intervention strategies to be targeted at such high-risk strains as soon as they begin to emerge. Automated systems could be used to highlight such clades for further epidemiological investigation.

For molecular epidemiology and in surveillance of the antimicrobial resistance of gonococci nationally and internationally, WGS should soon be the method of choice. WGS becomes more cost-effective every year, and platforms such as WGSA provide automated analysis of WGS data linked to patient metadata. However, appropriate funding, capacity building, and quality assurance remain essential for use of WGS in national and international surveillance of gonococci.
