# Supplementary material for: Public health surveillance of multidrug-resistant clones of Neisseria gonorrhoeae in Europe: a genomic survey
Source: Lancet Infect Dis. 2018 Jul;18(7):758–68. doi: 10.1016/S1473-3099(18)30225-1 (PMC6010626; doi:10.1016/S1473-3099(18)30225-1)
Supplement: Supplementary appendix 1 [file mmc1.pdf]

THE LANCET Infectious Diseases

Supplementary appendix

This appendix formed part of the original submission and has been peer reviewed. We post it as supplied by the authors.

Supplement to: Harris SR, Cole MJ, Spiteri G, et al, on behalf of the Euro-GASP study group. Public health surveillance of multidrug-resistant clones of *Neisseria gonorrhoeae* in Europe: a genomic survey. *Lancet Infect Dis* 2018; published online May 15. http://dx.doi.org/10.1016/S1473-3099(18)30225-1.

Public health surveillance of multidrug-resistant clones of *Neisseria gonorrhoeae* in Europe: a genomic survey

Simon R. Harris, Michelle J. Cole, Gianfranco Spiteri, Leonor Sánchez-Busó, Daniel Golparian, Susanne Jacobsson, Richard Goater, Khalil Abudahab, Corin A. Yeats, Beatrice Bercot, Maria José Borrego, Brendan Crowley, Paola Stefanelli, Francesco Tripodo, Raquel Abad, David M. Aanensen, Magnus Unemo, on behalf of the Euro-GASP study group

Supplementary Methods

Euro-GASP[1,2]

European Gonococcal Antimicrobial Surveillance Programme (Euro-GASP) laboratories submit consecutive isolates for centralised antimicrobial susceptibility testing or, if they have fulfilled set quality criteria, perform decentralised testing in their own laboratory. In 2013, Belgium, Denmark, France, Greece, Iceland, Italy, Malta, The Netherlands, Portugal, Spain, Sweden, and United Kingdom performed decentralised antimicrobial susceptibility testing, while Austria, Cyprus, Germany, Hungary, Latvia, Norway, Slovenia, and Slovakia were included in the centralised testing model. The centralised testing was performed at Public Health England, London, United Kingdom or at Örebro University Hospital, Örebro, Sweden. To ensure that data quality is maintained in decentralised testing in Euro-GASP, the following quality criteria have to be fulfilled to perform decentralised antimicrobial susceptibility testing: i) Laboratories have to perform consistently well in the mandatory external quality assessment (EQA) scheme: no more than 5% of MIC results should differ by more than two MIC doubling dilutions of the modal MICs, ii) Laboratories are required to demonstrate good comparability: at least 90% agreement between antimicrobial resistance (AMR) category, and no more than 5% of MIC results should differ by more than two MIC doubling dilutions between the laboratories own national or regional susceptibility testing data, and the susceptibility data generated by centralised susceptibility testing, and iii) for quality control, laboratories should test the *Neisseria gonorrhoeae* reference strains WHO G, WHO K, WHO M, WHO O, and WHO P[3] on the agar dilution plates and each batch of Etests. Furthermore, both laboratories performing decentralised and centralised antimicrobial susceptibility testing strictly follow the developed Euro-GASP protocols for testing.[1,2]

Whole genome sequencing

DNA extracts were multiplexed and sequenced on the Illumina HiSeq platform at the Wellcome Sanger Institute. Sequence reads were quality checked for median base quality >30, low levels of adapter contamination, consistent base content along the length of the reads, GC content consistent with a *N. gonorrhoeae* genome (~44% mean), mapping to >90% of the bases of the *N. gonorrhoeae* FA1090 reference genome sequence, and consistent mapping to parts of the reference genome with different GC contents. After passing quality control, reads were assembled using the Sanger Institute assembly pipeline,[4] with SPAdes v3.9.0[5,6] using the careful option, the coverage cut-off set to auto and kmer sizes from 41 to 109 and a step size of four. Contigs were strictly filtered to remove contaminating sequence by using Mash[7] to identify the best match for each contig against the RefSeq bacteria database release 78[8] augmented with the 2016 WHO gonococcal reference strain sequences.[3] Any contigs for which a gonococcal sequence was not the best match were excluded from further analysis. Potential mixed samples were flagged based on identifying the presence of multiple multi-locus sequence typing (MLST), NG-MAST (*porB*, *tbpB*), *porA* or *penA* alleles (all considered as single-copy genes) within a sample.

Detailed explanations of the methods employed in WGSA can be found at https://live.wgsa.net/documentation. Annotated genes from the 14 WHO reference genomes[3] were clustered using ROARY[9] to identify gene families. Families that produced overlapping matches on the references were trimmed or merged to remove overlaps >30 nucleotides and families with >1 match to a reference (e.g. paralogues) were removed. A strict definition of a core family was used, whereby the family would be required to be present in all of the 14 WHO reference genomes.[3] Using this approach, 1,710 core gene families were identified, equating to ~1.5 Mb, or ~70% of the mean genome length.

The assembly of each isolate was scanned with representative alleles from the gene families using BLASTN (default parameters, E-value ≤ $1e^{-35}$, percent identity >80). If two matches overlapped by >30 nucleotides, the shorter match was removed. Complete matches and incomplete matches with >80% of

the representative allele were kept if no complete match was found. All variant sites were then recorded for each allele.

The number of single nucleotide polymorphisms (SNPs) between core genes from each pair of assemblies was counted and the resulting matrix clustered into a dendrogram using the APE package from R, and mid-point rooted using Phangorn.

A curated set of known AMR determinants (Supplementary Table 1) were searched against the assemblies of the isolates using BLASTN under default parameters. Genes had to be 80% complete to be analysed further. AMR determinants were grouped into sets of one or more that collaboratively provide resistance to each antimicrobial. Complete sets indicate intermediate or full resistance predicted to the specified antimicrobial; incomplete sets may confer intermediate or no resistance.

A representative of each MLST locus was used to search the assembly with BLAST with the following arguments: -reward 1 -penalty -1 -gapopen 0 -gapextend 2. Highest scoring complete matches at each locus were then assigned MLST codes using identical alleles in the schema from https://pubmlst.org/neisseria/. If no identical allele was found, a WGSA code was assigned (e.g. WM1).

NG-MAST STs were assigned using NG-master[10] with data from the NG-MAST database (http://www.ng-mast.net). MLST STs were identified using the PubMLST database (http://pubmlst.org/neisseria). NG-MAST genogroups were mainly assigned as previously reported.[11] Briefly, different NG-MAST STs were assigned to a "genogroup", if one identical allele was shared and the other allele showed ≥99% similarity (≤5 bp difference for *porB* and ≤4 bp for *tbpB*) or the concatenated sequence of both alleles displayed ≥99.4% similarity to the concatenated sequence of both alleles of the main ST in the genogroup. Genogroups were named with "G" followed by the number of isolates with the predominant ST within each genogroup (Supplementary Table 2).

Consistency and retention indices (CI and RI, respectively) were calculated by comparing the tree topology with the observed distribution of NG-MAST STs and genogroups and MLST STs using the dendropy python package.[12] The CI is a measure of relative homoplasy, calculated as the ratio of the minimum number of possible state changes given the input data to the observed number of state changes on the tree. The RI is a measure of how much expected synapomorphy is observed in the data.

Statistical analysis

Associations between NG-MAST genogroups, AMR, and patient characteristics (gender, age, and sexual orientation) were examined using the pandas,[13] numpy,[14] scipy,[15] and statsmodel[16] modules of python3. For discrete predictors where datasets contained sufficient numbers, crude odds ratios (OR) and 95% confidence intervals (CI) were calculated using scipy.[15] A Pearson χ^2-test was used to test if these ORs were significantly different from 1. In cases with small or zero cell numbers, Fisher's exact test was performed. Statistical significance was assumed when $p<0{\cdot}05$. For continuous predictors logistic regression was performed using statsmodel.[16] Tests were carried out excluding missing data, except when explicitly testing for association with missing data. For analyses of MIC values, data was log2 transformed prior to analysis.

Detection of clades linked to changes in phenotype distribution

Changes in phenotype distribution were assessed using TreeBreaker.[17] For each run, isolates were pruned from the tree if their phenotype was unknown, and the algorithm was run for 10,000,000 generations, including a burn-in of 5,000,000 generations. In all cases, changes of phenotype distribution were accepted if the posterior on a branch was ≥0·5 and included at least 5 isolates.

Supplementary Figures

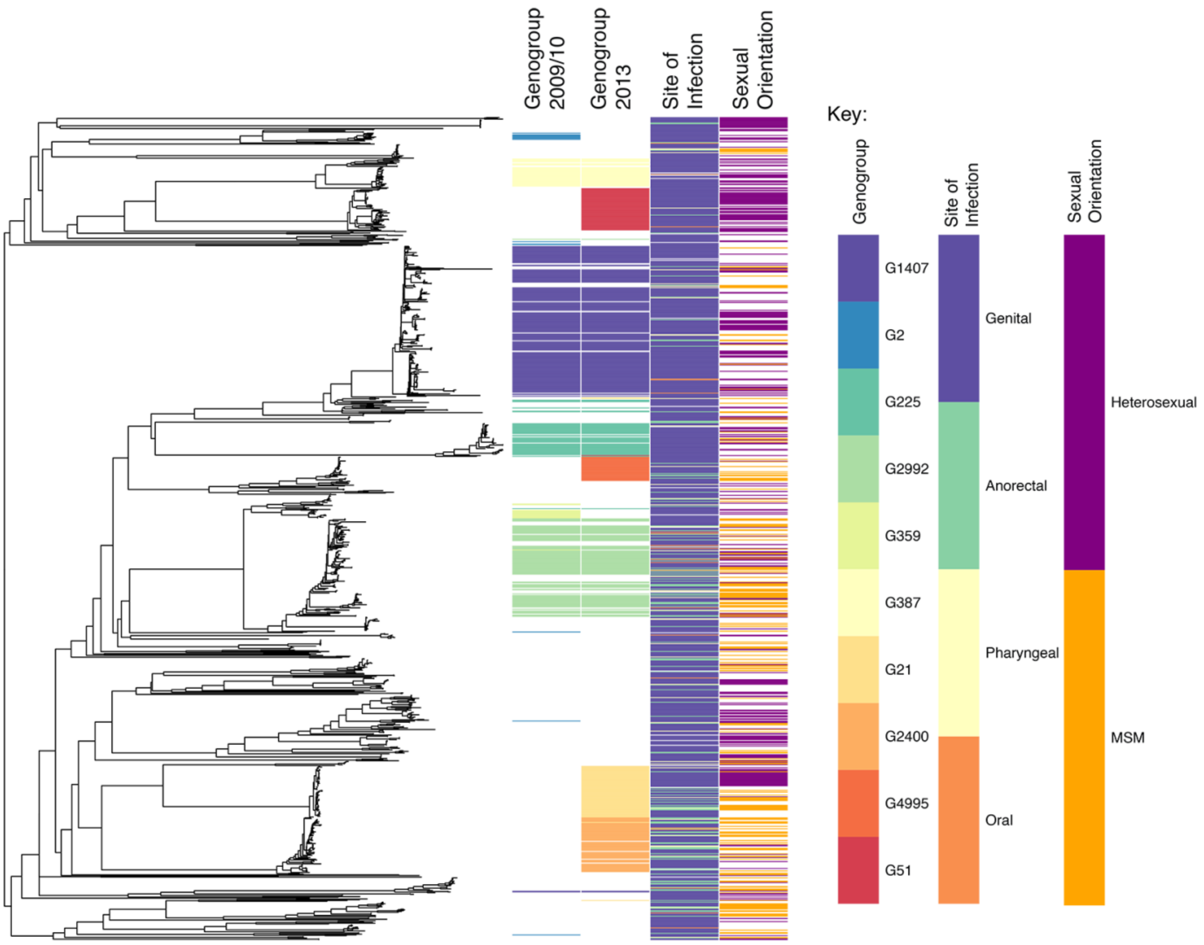

Supplementary Figure 1. **The changing population structure of *Neisseria gonorrhoeae* in the European Union/European Economic Area (EU/EEA).** On the left of the figure is the Whole Genome Sequence Analysis (WGSA; www.wgsa.net) phylogenetic reconstruction of the 1054 isolates whole genome sequenced from the Euro-GASP 2013 collection. Next to the tree are displayed columns indicating isolates in the most prevalent *N. gonorrhoeae* multi-antigen sequence typing (NG-MAST) genogroups in the 2009-2010 survey[11] and the present 2013 survey and clinical information on site of infection and sexual orientation. NG-MAST genogroups that were not common in 2009-2010,[11] but had become more prevalent in 2013 are relatively tightly clustered on long branches, suggestive of recent introduction into the region. Figure produced using Phandango.[18]

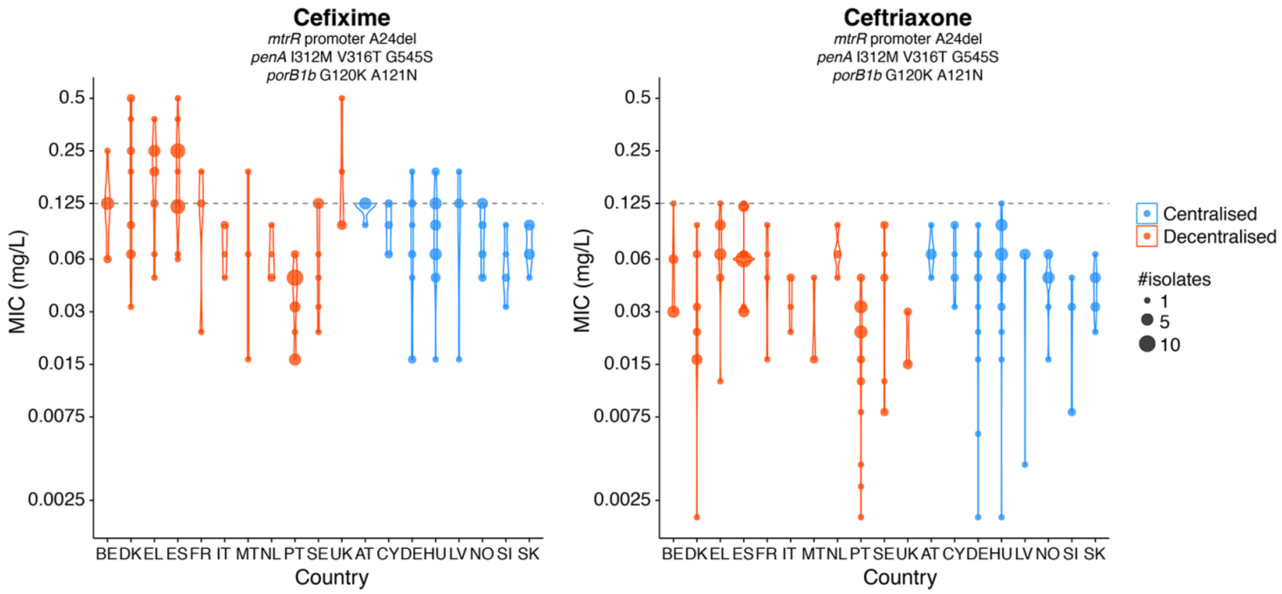

Supplementary Figure 2. Violin plots of observed minimum inhibitory concentrations (after retesting; mg/L) per country for cefixime and ceftriaxone for all isolates with a deletion of an A in the repeat sequence in the promoter of *mtrR*, I312M, V316T and G545S substitutions in *penA* and G120K and A121 substitutions in *porB1b*. Dashed horizontal lines indicate EUCAST breakpoints (www.eucast.org). Countries for whom testing was carried out centrally are shown in blue, and those using decentralised testing are in red. BE, Belgium; DK, Denmark; EL, Greece; ES, Spain; FR, France; IT, Italy; MT, Malta; NL, The Netherlands; PT, Portugal; SE, Sweden; UK, United Kingdom; AT, Austria; CY, Cyprus; DE, Germany; HU, Hungary; LV, Latvia; NO, Norway; SI, Slovenia: SK, Slovakia

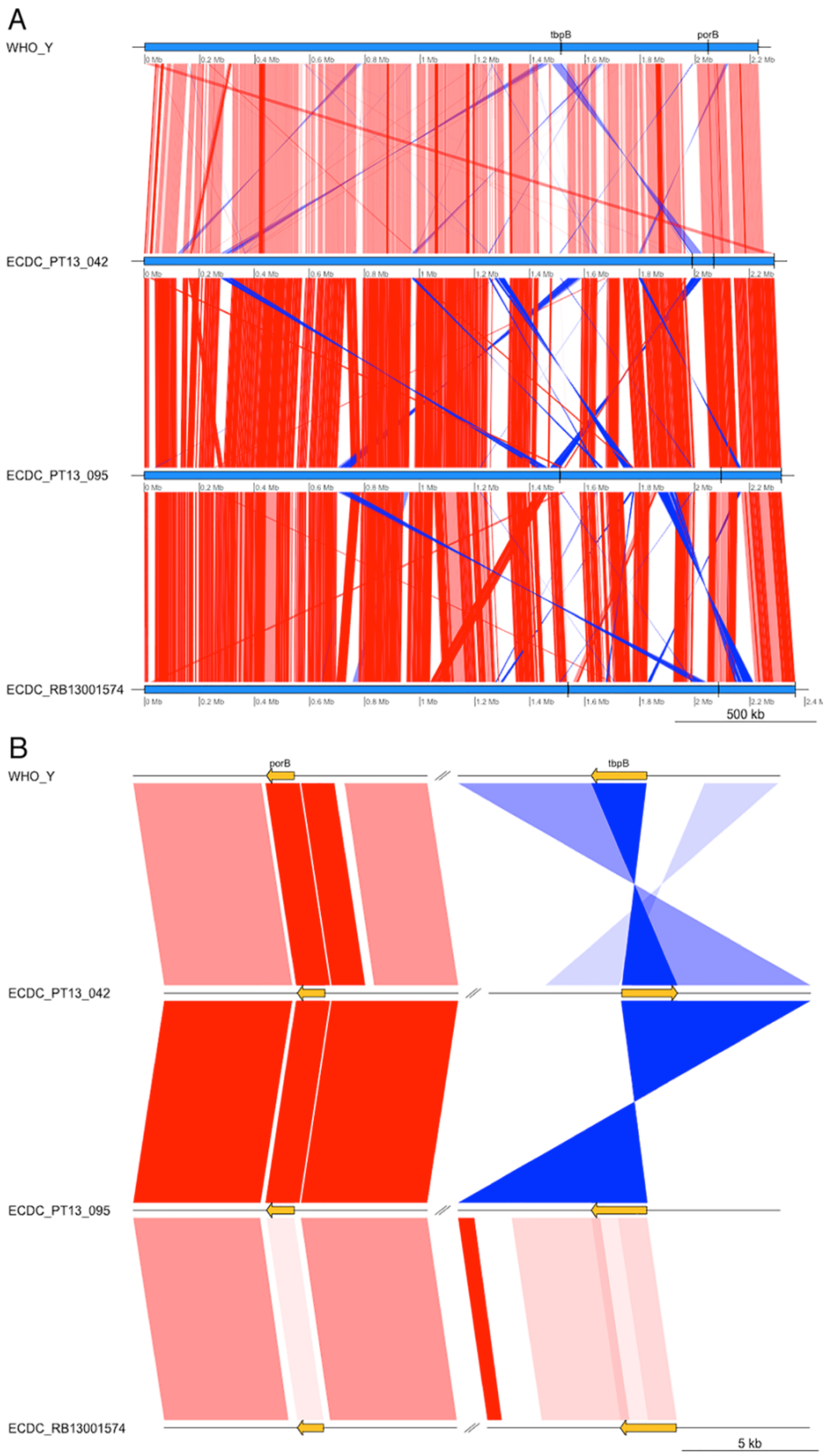

Supplementary Figure 3. **Genome comparisons of *Neisseria gonorrhoeae* multi-antigen sequence typing (NG-MAST) ST1407 isolates, illustrating NG-MAST convergence caused by recombination affecting the two NG-MAST loci.** A) Genome comparison of ST1407 isolates showing that the two recombinant isolates are highly dissimilar to WHO Y[3] across most of their genome. Each blue row indicates the genome of an isolate. Where genomes were not complete, contigs were ordered relative to the complete WHO Y reference. Coloured bars joining adjacent genomes indicate BLAST matches. Red = matches on the same strand; blue = matches on opposite strands. The intensity of the colour of the matches indicates how strong the match is. Top is the WHO Y reference genome, which is an example of the canonical ST 1407 clade. The two middle isolates are both ST1407, but phylogenetically unrelated based on WGS data. The bottom isolate is not ST1407, but is the closest match to the 1407 recombinants in the WGS tree. The position of the two NG-MAST loci, *porB* and *tbpB* are indicated on each genome. B) Zoomed comparison of the region around the two NG-MAST loci, *porB* and *tbpB* showing the recombinations that have occurred at these loci.

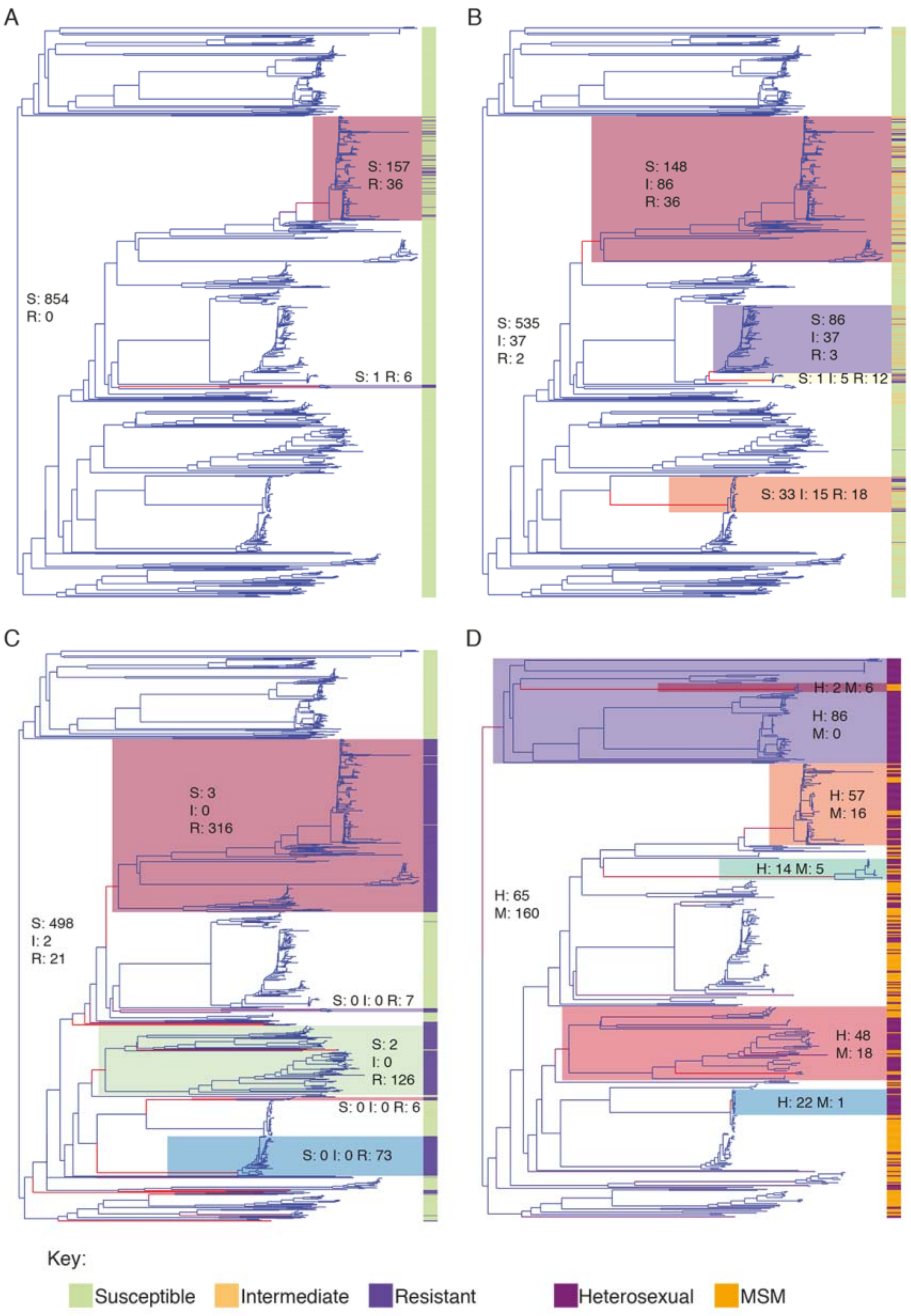

Supplementary Figure 4. **TreeBreaker[17] identified changes in distributions of variables across the whole genome sequencing (WGS) phylogeny.** Coloured boxes on the tree indicate clades with different distributions of a metadata variable. Particular colours have no specific meaning and are not linked between panels. The distributions within each coloured clade are indicated on the tree, as well as the distribution for the background population (area in white). To the right of each tree is a graphical representation of the distribution of the variable in the isolates, with the colour key for this shown at the bottom of the figure. A) Cefixime susceptible (S), intermediate (I), and resistant (R); B) Azithromycin S, I, and R; C) Ciprofloxacin S, I, and R; and D) Sexual orientation. SIR classification was performed according to EUCAST breakpoints (www.eucast.org).

Supplementary Table Legends

Supplementary Table 1. **Genotypic determinants used by WGSA (www.wgsa.net) to predict decreased susceptibility and resistance to antimicrobials.** AZM = azithromycin; CFM = cefixime; CRO = ceftriaxone; PEN = penicillin G; TET = tetracycline; DOX = doxycycline; CIP = ciprofloxacin; SPT = spectinomycin; SMX = sulphamethoxazole; WGSA=Whole Genome Sequence Analysis web analysis tool.

Supplementary Table 2. **NG-MAST STs comprising each NG-MAST genogroup in this study.** Genogroups were mainly assigned as previously reported.[11]

Supplementary Table 3. **Patient demographics and antimicrobial phenotypes of isolates (after retesting of discrepant isolates) belonging to the top eight NG-MAST genogroups in the 2013 Euro-GASP collection.** NG-MAST = *N. gonorrhoeae* multi-antigen sequence typing; MSM = men-who-have-sex-with-men; AZM = azithromycin; CIP = ciprofloxacin; CFM = cefixime; S = susceptible; I = intermediate; R = resistant. For gender, sexual orientation, AZM, CIP and CFM, numbers indicate the percentage of isolates in the genogroup belonging with each variable value. Numbers in brackets indicate the percentage of isolates with each variable value which are in the genogroup. For example, for male gender, the number indicates the percentage of the isolates in the genogroup which were isolated from a male patient, while the number in brackets indicates the percentage of isolates from males which were in the genogroup.

Supplementary Table 4. **Odds ratios for patient demographics and antimicrobial phenotypes of isolates in the top eight NG-MAST genogroups and mosaic *penA*-associated MLSTs and WGS clades in the 2013 Euro-GASP collection.** Numbers in brackets following p-values indicate the statistical test employed. 1 = Chi-squared, 2 = Fisher exact and 3 = logistic regression. OR = odds ratio, with confidence intervals in brackets; p = p value; NG-MAST = *N. gonorrhoeae* multiantigen sequence typing; MSM = men-who-have-sex-with-men; AZM = azithromycin; CIP = ciprofloxacin; CFM = cefixime; S = susceptible; I = intermediate; R = resistant; MIC = minimum inhibitory concentration. For logistic regressions, age and MIC were used as the predicting variables and NG-MAST genogroup, MLST ST or phylogenetic clade as the outcome. The OR for age is expressed for increasing age. The OR for MIC is expressed for increasing log MIC.

References

1. European Centre for Disease Prevention and Control. Gonococcal antimicrobial susceptibility surveillance in Europe 2015. Stockholm, Sweden: ECDC; 2017. https://ecdc.europa.eu/sites/portal/files/documents/gonococcal-antimicrobial-susceptibility-surveillance-Europe-2015.pdf (accessed Feb 5, 2018).
2. European Centre for Disease Prevention and Control. Gonococcal antimicrobial susceptibility surveillance in Europe 2013. Stockholm, Sweden: ECDC; 2015. https://ecdc.europa.eu/sites/portal/files/media/en/publications/Publications/gonococcal-antimicrobial-susceptibility-surveillance-europe-2013.pdf (accessed Feb 5, 2018).
3. Unemo M, Golparian D, Sánchez-Busó L, et al. The novel 2016 WHO *Neisseria gonorrhoeae* reference strains for global quality assurance of laboratory investigations: phenotypic, genetic and reference genome characterization. *J Antimicrob Chemother* 2016; **71**: 3096-108.
4. Page AJ, De Silva N, Hunt M, et al. Robust high-throughput prokaryote de novo assembly and improvement pipeline for Illumina data. *Microb Genom* 2016; **2**: e000083.
5. Nurk S, Bankevich A, Antipov D, et al. Assembling single-cell genomes and mini-metagenomes from chimeric MDA products. *J Comput Biol* 2013; **20**: 714-37.
6. Nurk S, Bankevich A, Antipov D, et al. Assembling genomes and mini-metagenomes from highly chimeric reads. In: Lecture Notes in Computer Science. 2013: 158–70.
7. Ondov BD, Treangen TJ, Melsted P, et al. Mash: fast genome and metagenome distance estimation using MinHash. *Genome Biol* 2016; **17**: 132.
8. O'Leary NA, Wright MW, Brister JR, et al. Reference sequence (RefSeq) database at NCBI: current status, taxonomic expansion, and functional annotation. *Nucleic Acids Res* 2016; **44**: D733-45.
9. Page AJ, Cummins CA, Hunt M, et al. Roary: rapid large-scale prokaryote pan genome analysis. *Bioinformatics* 2015; **31**: 3691-3.
10. Kwong JC, Gonçalves da Silva A, Dyet K, et al. NGMASTER: in silico multi-antigen sequence typing for *Neisseria gonorrhoeae*. *Microbial Genomics* 2016; **2**: e000076.
11. Chisholm SA, Unemo M, Quaye N, et al. Molecular epidemiological typing within the European Gonococcal Antimicrobial Resistance Surveillance Programme reveals predominance of a multidrug-resistant clone. *Euro Surveill* 2013; **18**(3).
12. Sukumaran J, Holder MT. DendroPy: a Python library for phylogenetic computing. *Bioinformatics* 2010; **26**: 1569–71.

13. McKinney W. Data structures for statistical computing in Python. In: van der Walt S, Millman J, eds. Proceedings of the 9th Python in Science Conference. 2010: 51–56.
14. van der Walt S, Chris Colbert S, Varoquaux G. The NumPy Array: A structure for efficient numerical computation. *Comput Sci Eng* 2011; **13**: 22–30.
15. Jones E, Oliphant T, Peterson P, et al. SciPy: Open source scientific tools for Python. 2001. http://www.scipy.org/ (accessed Feb 5, 2018).
16. Seabold S, Perktold J. Statsmodels: Econometric and statistical modeling with python. In: 9th Python in Science Conference. 2010.
17. Ansari MA, Didelot X. Bayesian inference of the evolution of a phenotype distribution on a phylogenetic tree. *Genetics* 2016; **204**: 89-98.
18. Hadfield J, Croucher NJ, Goater RJ, Abudahab K, Aanensen DM, Harris SR. Phandango: an interactive viewer for bacterial population genomics. 2017 Sep 25. doi: 10.1093/bioinformatics/btx610. [Epub ahead of print]
